# Antiretroviral Pre-exposure Prophylaxis Prevents Vaginal Transmission of HIV-1 in Humanized BLT Mice

**DOI:** 10.1371/journal.pmed.0050016

**Published:** 2008-01-15

**Authors:** Paul W Denton, Jacob D Estes, Zhifeng Sun, Florence A Othieno, Bangdong L Wei, Anja K Wege, Daniel A Powell, Deborah Payne, Ashley T Haase, J. Victor Garcia

**Affiliations:** 1 Department of Internal Medicine, Division of Infectious Diseases, University of Texas Southwestern Medical Center at Dallas, Dallas, Texas, United States of America; 2 Department of Microbiology, Medical School, University of Minnesota, Minneapolis, Minnesota, United States of America; 3 Department of Pathology, University of Texas Southwestern Medical Center at Dallas, Dallas, Texas, United States of America; University of California Davis, United States of America

## Abstract

**Background:**

Worldwide, vaginal transmission now accounts for more than half of newly acquired HIV-1 infections. Despite the urgency to develop and implement novel approaches capable of preventing HIV transmission, this process has been hindered by the lack of adequate small animal models for preclinical efficacy and safety testing. Given the importance of this route of transmission, we investigated the susceptibility of humanized mice to intravaginal HIV-1 infection.

**Methods and Findings:**

We show that the female reproductive tract of humanized bone marrow–liver–thymus (BLT) mice is reconstituted with human CD4^+^ T and other relevant human cells, rendering these humanized mice susceptible to intravaginal infection by HIV-1. Effects of HIV-1 infection include CD4^+^ T cell depletion in gut-associated lymphoid tissue (GALT) that closely mimics what is observed in HIV-1–infected humans. We also show that pre-exposure prophylaxis with antiretroviral drugs is a highly effective method for preventing vaginal HIV-1 transmission. Whereas 88% (7/8) of BLT mice inoculated vaginally with HIV-1 became infected, none of the animals (0/5) given pre-exposure prophylaxis of emtricitabine (FTC)/tenofovir disoproxil fumarate (TDF) showed evidence of infection (Chi square = 7.5, *df* = 1, *p* = 0.006).

**Conclusions:**

The fact that humanized BLT mice are susceptible to intravaginal infection makes this system an excellent candidate for preclinical evaluation of both microbicides and pre-exposure prophylactic regimens. The utility of humanized mice to study intravaginal HIV-1 transmission is particularly highlighted by the demonstration that pre-exposure prophylaxis can prevent intravaginal HIV-1 transmission in the BLT mouse model.

## Abstract 2

J. Victor Garcia and colleagues show that mice with immune systems reconstituted from human bone marrow, liver, and thymus transplants provide a model for prevention of intravaginal HIV infection.

## Editors' Summary

**Background.:**

Since the first cases of acquired immunodeficiency syndrome (AIDS) in 1981, the AIDS epidemic has spread rapidly. About 33 million people are now infected with the human immunodeficiency virus (HIV), the cause of AIDS. More than half of newly acquired infections now occur in women, mostly through unprotected vaginal sex with an infected male partner. Women are biologically more susceptible than men to HIV infection during vaginal intercourse and often cannot persuade their partner to use a condom. Consequently, alternative strategies that prevent intravaginal transmission of HIV (infection through the vagina) are urgently needed, particularly strategies that women can use without their partner's agreement. A vaccine would be ideal but it could be many years before an effective HIV vaccine is available so researchers are investigating other preventative strategies such as the use of microbicides—compounds that protect against HIV when applied inside the vagina—and pre-exposure treatment (prophylaxis) with antiretroviral drugs.

**Why Was This Study Done?:**

Before any new strategy to prevent intravaginal HIV transmission is tried by women, it has to be tested in animals. Currently, this can only be done in macaques, an expensive option. In this study, the researchers have investigated whether “humanized BLT” mice could be used instead. When HIV enters the human body during vaginal intercourse, it sticks to dendritic cells (a type of immune system cell) in the vaginal lining. These cells carry the virus to the body's lymphoid tissues (collections of immune cells), where it infects and kills CD4^+^ T cells (another type of immune cell). Dendritic cells and CD4^+^ T cells have molecules on their surface that HIV recognizes. Mice are not normally susceptible to infection with HIV because their immune system cells lack these molecules. Humanized BLT mice have a nearly human immune system—BLT stands for bone marrow, liver, thymus. They are produced by implanting pieces of human fetal liver and thymus (the organ where T cells learn to recognize foreign invaders) under the kidney capsule of immunodeficient mice (animals born without an immune system) and then transplanting human hematopoietic stem cells (the source of the major immune system cells) into the mice.

**What Did the Researchers Do and Find?:**

When the researchers examined the female reproductive tract of humanized BLT mice for human immune system cells, they found CD4^+^ T cells, dendritic cells and macrophages, all of which are involved in HIV infection. Furthermore, half of the blood cells of the BLT mice were human. Most of the BLT mice, the researchers report, were susceptible to intravaginal HIV infection as shown, for example, by a rapid loss of human CD4^+^ T cells from their blood. However, BLT mice pretreated with antiretroviral drugs (a mixture of emtricitabine and tenofovir disoproxil fumarate) were resistant to intravaginal HIV infection. As in human HIV infections, CD4^+^ T cells were also depleted in several other organs of the BLT mice after intravaginal HIV infection. Again, this depletion was prevented by antiretroviral pre-exposure prophylaxis. Finally, human CD4^+^ T cells also disappeared from the gut-associated lymphoid tissue (an important site for HIV replication and CD4^+^ T cell depletion during human HIV disease) of the BLT mice after infection with HIV.

**What Do These Findings Mean?:**

These findings show that humanized BLT mice are susceptible to intravaginal infection with HIV and that many aspects of HIV infection in these mice closely mimic infection in people. In addition, by showing that pre-exposure prophylaxis with antiretroviral drugs prevents HIV infection, these results suggest that humanized BLT mice could be used to test new strategies designed to prevent intravaginal infection. As with all animal models, any approach that works in humanized BLT mice will still have to be tested in people. Nevertheless, these findings provide preclinical evidence that pre-exposure prophylaxis with antiretroviral drugs may be an effective way to prevent intravaginal transmission of HIV and, therefore, provide valuable support for clinical trials of this approach.

**Additional Information.:**

Please access these Web sites via the online version of this summary at http://dx.doi.org/10.1371/journal.pmed.0050016.

- Information is available from the US National Institute of Allergy and Infectious Diseases on HIV infection and AIDS and on HIV infection in women
- HIVInSite has comprehensive information on all aspects of HIV/AIDS, including articles on women and HIV and on safer sex, which includes information on pre-exposure prophylaxis and microbicides
- Information is available from Avert, an international AIDS charity, on HIV prevention, on women, HIV, and AIDS, and on microbicides
- The US Centers for Disease Control and Prevention provides information on HIV/AIDS, including information on HIV/AIDS among women and on CDC trials of pre-exposure prophylaxis for HIV prevention (in English and some information in Spanish)
- PrEP Watch is a comprehensive information source on pre-exposure prophylaxis for HIV prevention

## Introduction

HIV, the causative agent of AIDS, is predominantly transmitted by unprotected sexual contact [1]. Currently, women worldwide account for more than half of the estimated 6,800 newly acquired infections every day, with a majority of those transmissions occurring via the vaginal route [1]. Therefore, it is critical that strategies to prevent vaginal transmission of HIV be developed and implemented.

Without an effective vaccine on the horizon, alternative strategies are urgently needed to prevent the further spread of AIDS. Development and efficacy testing of microbicides and other preventive strategies, such as antiretroviral pre-exposure prophylaxis, necessitate animal models [2–5]. Currently, the only surrogate animal model used to study intravaginal HIV transmission are macaques infected with simian immunodeficiency virus (SIV) or SIV/HIV (SHIV) chimeric viruses [6,7]. This model recapitulates several aspects of human infection, but does not support HIV-1 replication. In addition, the expense of non-human primate experiments is high, the availability of animals (especially females) is limited, and variations in host susceptibility along with disease progression occur because non-human primate colonies are outbred. Consequently, there is significant need to develop animal models to investigate measures to prevent intravaginal HIV-1 transmission.

Using humanized mice to address this need is attractive because they allow in vivo studies of HIV-1 infection of human cells. In addition, in vivo preclinical evaluation of potential clinical interventions can minimize human risk [8]. A common aspect of producing humanized mice is transplanting human hematopoietic stem cells (HSC) into one of several immunodeficient mouse strains (reviewed in [8]). The mouse age at transplant and anatomical locations of the transplants vary by model, but in each system, the transplanted HSC engraft the mouse bone marrow. These engrafted HSC then differentiate in situ into human hematopoietic lineages including T, B, myeloid, and dendritic cells [9,10]. It should be noted that in this context, human thymocytes are generated in the mouse thymus and in the absence of human thymic stroma. Alternatively, human thymocytes can develop in the context of human thymus in the SCID-hu thy/liv mouse model [11,12], which has been used to evaluate the efficacy of antiretrovirals, including emtricitabine (FTC), efavirenz, atazanavir, and enfuvirtide [13]. The SCID-hu thy/liv model does not incorporate transplantation of human HSC into the recipient mouse; it involves only implantation of human fetal thymus and liver beneath the kidney capsule of SCID mice. A human thymus complete with human thymocytes is generated, yet systemic reconstitution of SCID-hu thy/liv mice with human hematopoietic cells is sparse and limited to T cells [14].

Humanized bone marrow–liver–thymus (BLT) mice [15] represent advancement beyond these models. Humanized BLT mice are generated by initially implanting human fetal liver and thymus tissue under the kidney capsule of an immunodeficient mouse (as with SCID-hu thy/liv), followed by an autologous human HSC transplant of human fetal liver CD34^+^ cells (similar to other humanization protocols). Thus, humanized BLT mice combine the most desirable attributes of other humanized mouse models into a single system. Namely, in humanized BLT mice, there is human thymic tissue where T cell education occurs, and there is complete systemic reconstitution of all major human hematopoietic lineages, including T, B, monocyte/macrophage, dendritic, and natural killer cells [15]. In addition, BLT mice have been shown to mount robust human immune responses, such as antigen-specific human immunoglobulin G (IgG) production [16] and xenograft rejection [17]. Human T cells in BLT mice can generate human leukocyte antigen class I– and class II–restricted adaptive immune responses to Epstein-Barr virus and are activated by human dendritic cells to mount a potent T cell immune response to superantigens [15]. Particularly relevant to this study is the extensive reconstitution of lymphoid tissue within the gut of humanized BLT mice [15,16]. With this particular finding in mind, we hypothesized that mucosal reconstitution with human lymphoid cells would be systemic and would include the female reproductive tract (FRT), rendering female BLT mice susceptible to intravaginal HIV-1 infection.

To conclusively address these hypotheses, we designed the present study. Our aims were to (1) characterize the reconstitution of the FRT with human lymphoid cells; (2) test the susceptibility of humanized BLT mice to viral transmission following a single intravaginal exposure to cell-free HIV-1; (3) characterize systemic pathogenic effects of HIV-1 transmitted intravaginally and disseminated throughout humanized BLT mice, including effects in the gut-associated lymphoid tissue (GALT); and (4) utilize this small animal model to conduct pre-clinical evaluation of antiretroviral pre-exposure prophylaxis for intravaginal HIV-1 transmission.

## Materials and Methods

### Preparation of Humanized BLT Mice, Tissue Harvesting, and Microscopic and Flow Cytometric Analyses

Humanized BLT mice were prepared essentially as we have previously described [15,16]. Briefly, thy/liv-implanted mice [12] were transplanted with autologous human fetal liver CD34^+^ cells (Advanced Bioscience Resources) and monitored for human reconstitution in peripheral blood by flow cytometry as we have previously described [15,16]. Mice were maintained at the Animal Resources Center of University of Texas Southwestern Medical Center (UTSWMC) in accordance with protocols approved by the UTSWMC Institutional Animal Care and Use Committee. Tissues were harvested for both microscopic and flow cytometric analyses. Immunohistochemical and in situ analyses were performed essentially as previously described [15,16]. Specific controls for immunohistochemistry included staining tissue sections from humanized BLT mice with isotype-matched negative control antibodies (mouse IgG1, mouse IgG2a, goat ChromPure IgG, and rabbit ChromPure IgG) to demonstrate that appropriate human lineages were being detected. Conversely, mice never reconstituted with human cells were stained with anti-human CD3, CD4, CD68, and CD11c to rule out the staining of any non-human cells by these antibodies (unpublished data; [15,16]). Single-cell suspensions for flow cytometric analysis of each tissue were prepared essentially as we have previously described [15,16].

### Intravaginal Exposure of Humanized BLT Mice to HIV-1

Stocks of HIV-1_JR-CSF_ [18] were prepared, titered, and p24 content determined as we have previously described [19,20]. Prior to inoculation, mice were anesthetized with sodium pentobarbital. Atraumatic intravaginal inoculations were performed essentially as previously described [21] using a total volume of 10 μl (170 ng of p24, ∼9 × 10^4^ tissue culture infectious units). FTC and tenofovir disoproxil fumarate (TDF) (Gilead) were administered intraperitonealy (3.5 mg and 5.2 mg, respectively) once daily for seven consecutive days starting 48 h prior to intravaginal inoculation with HIV-1 [13,22,23].

### Analysis of HIV Infection of Humanized BLT Mice

Infection of BLT mice with HIV was monitored in peripheral blood by determining plasma viral load (Amplicor; Roche), plasma levels of viral antigenemia (ELISA p24 [sensitivity: 12 pg/ml of diluted mouse plasma]; Coulter) and by determining the levels of human CD4^+^ and CD8^+^ T cells in peripheral blood (flow cytometry) essentially as we have previously described [15,16,20]. Analysis of systemic infection was performed by in situ hybridization and flow cytometry, also as we have previously described [15,16,24]. Quantitative real-time PCR for viral DNA was performed using Assays-on-Demand in a 7500 Fast instrument (sensitivity: five copies of JR-CSF; SDS software version 1.3.1.22; Applied Biosystems) following the manufacturer's protocol for universal cycling conditions. ABI custom TaqMan reagents were: Forward primer: 5′-ATCAAGCAGCTATGCAAATGCT-3′; Reverse primer: 3′-CTGAAGGGTACTAGTAGTCCCTGCTATGTC-5′; and MGB probe: 5′-TCAATGAGGAAGCTGCAGAA-3′. Rescue of infectious virus from the indicated tissues was performed by coculture with PHA/IL2-activated PBMC from HIV seronegative donors, and viral spread was monitored by determining p24 levels in the culture supernatant [16]. Human CD4^+^ T cell depletion was monitored in all tissues indicated essentially as we have previously described using six-color flow cytometry (FACSCanto; BD Biosciences) with analysis performed in FACSDiva software (BD Biosciences) [16]. Statistical analysis using the Student *t* test and the Kaplan-Meier plot were performed using Prism v. 4 (Graph Pad Software).

## Results

**Figure 1:**
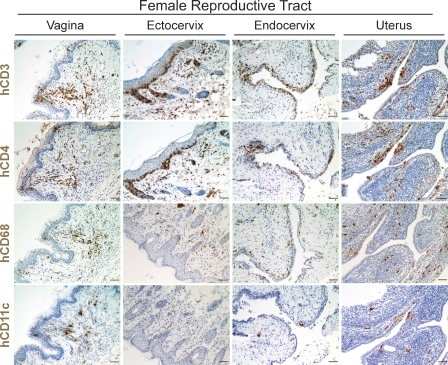
Reconstitution of the Female Reproductive Tract of Humanized BLT Mice with Human Hematopoietic Cells Immunohistochemical analysis of the vagina, ectocervix, endocervix, and uterus of female BLT mice for the presence of human hematopoietic lineages (brown cells) (bars indicate 25 μm). Robust reconstitution with cells relevant to HIV-1 infection, including human T cells, monocyte/macrophages, and dendritic cells, was observed in each compartment of the FRT of humanized BLT mice, demonstrating the efficient repopulation of these important mucosal sites.

The FRT represents a highly specialized and complex anatomical site where initial infection occurs following intravaginal exposure [25–27]. Therefore, we used immunohistochemistry to determine whether human lymphocytes and other cells important for HIV-1 infection were present in the vagina, ectocervix, endocervix, and uterus after reconstitution of BLT mice with human HSC. All populations of human cells necessary for HIV-1 infection (CD4^+^ T cells, macrophages, and dendritic cells) were found to be abundant throughout the FRT of BLT mice (Figure 1). Specifically, human CD4^+^ cells were distributed throughout the FRT. Also, human CD68^+^ monocyte/macrophage cells and clusters of human CD11c^+^ dendritic cells were identified throughout the FRT. Together, these data establish that in situ differentiation of human HSC leads to reconstitution of the FRT of BLT mice with the human hematopoietic cells relevant to mucosal HIV-1 transmission [28–30].

**Figure 2:**
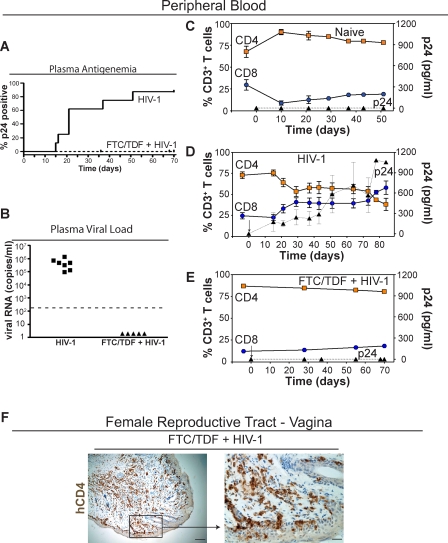
Pre-exposure Prophylaxis Prevents Intravaginal HIV-1 Transmission in Humanized BLT Mice (A) Kaplan-Meier plot of the time course to plasma antigenemia conversion following intravaginal HIV-1 exposure in BLT mice with or without the 7-d pre-exposure regimen of FTC/TDF (administered once daily starting 48 h prior to intravaginal inoculation). (B) Plasma from the seven infected BLT mice and the five FTC/TDF + HIV-1 mice was tested for the presence of HIV-1 RNA. Data presented depict the initial positive viral RNA value for each mouse examined. The dashed line indicates the limit of detection for this assay. (C–E) Shown are the levels of human CD4^+^ (orange squares) and CD8^+^ (blue circles) T cells in peripheral blood as well as the levels of HIV p24 antigenemia (black triangles) in plasma for (C) naive control (*n* = 6), (D) HIV-1 infected (*n* = 7), and (E) pre-exposure FTC/TDF-treated animals (*n* = 5). In (D), note that in seven of eight tested BLT mice, a single exposure to HIV-1 led to intravaginal transmission and an initial drop, with subsequent stabilization, in the levels of peripheral blood CD4^+^ human T cells. In contrast, no changes were observed in either the naive control (C) or BLT mice that received FTC/TDF for pre-exposure prophylaxis (E). In (D) and (E), day 0 is the day of inoculation and is indicated by an arrow. Gating strategy for flow cytometric analysis: live cells → human CD45 → human CD3 → human CD4 or CD8. (F) Immunohistochemical staining for human CD4^+^ cells within the vagina of a representative FTC/TDF-treated mouse demonstrating the continued presence of CD4^+^ T cells in this tissue (left, bar indicates 50 μm; right, bar indicates 12.5 μm; box indicates region magnified in subsequent image).

We then tested the susceptibility of humanized BLT mice to transmission of HIV-1 administered intravaginally. Prior to HIV-1 exposure, we analyzed the peripheral blood of all humanized BLT mice to be used in our study (8 to 12 wk post-transplant) and determined that, on average, slightly more than half (51.9% ± 7.2%) of all circulating peripheral blood cells were of human origin. We inoculated BLT mice (*n* = 8) with a single dose of cell-free HIV-1 (CCR5-tropic primary isolate JR-CSF [18]). BLT mice that did not receive HIV-1 (*n* = 6) were used as naive controls. In addition, we assessed intravaginal HIV-1 transmission in BLT mice administered a 7-d course of antiretroviral drugs (*n* = 5). We used emtricitabine and tenofovir disoproxil fumarate (FTC/TDF) because of potency, daily dosing, and favorable profiles for both toxicity and viral resistance [31]. FTC/TDF was administered 2 d prior to intravaginal inoculation, 3 h prior to inoculation, and for 4 d postinoculation. Whereas 88% (7/8) of BLT mice inoculated with HIV-1 became infected (Figure 2A: Chi square = 7.5, *df* = 1, *p* = 0.006), none of the animals (0/5) that received FTC/TDF showed evidence of infection (Figure 2A and 2B).

Neither naive nor FTC/TDF-treated BLT mice showed any evidence of plasma antigenemia (Figure 2C and 2E). In contrast, HIV-1 antigenemia was evident in the plasma from 7/8 intravaginally inoculated BLT mice as early as 2 wk postinfection (Figure 2D). Infection was corroborated by determining the viral load in the plasma of infected BLT mice. On average, 5.0 × 10^5^ (±1.5 × 10^5^) copies of RNA were detected per milliliter of plasma from the infected mice (Figure 2B). The appearance of HIV-1 in the plasma of infected mice preceded or coincided with a decline in peripheral blood human CD4^+^ T cells (Figure 2D). The levels of CD4^+^ T cells dropped by 30% during the first 3 wk postinfection and remained relatively constant for 7 wk, at which point there was a further 20% decline and an inversion of the ratio of CD4/CD8 cells (Figure 2D). Parallel to the decline of CD4^+^ T cells, there was an increase in the percentage of human CD8^+^ T cells in the periphery of infected BLT mice, which by 11 wk postinfection represented 60% of all the CD3^+^ cells in the periphery (Figure 2D). To eliminate the possibility that the lack of HIV-1 infection in FTC/TDF-treated mice resulted from a deficiency of cells that could be infected by HIV-1 within the mucosal portal of entry; the FRT of FTC/TDF-treated mice were examined for human CD4^+^ cells. The presence of CD4^+^ human cells in the vagina of inoculated mice that received pre-exposure prophylaxis with FTC/TDF rules out a lack of hematopoietic reconstitution of the FRT as responsible for the lack of infection (Figure 2F). Together, these results demonstrate the striking susceptibility of BLT mice to infection by HIV-1 administered intravaginally and highlight the extensive similarity in the course of HIV-1 infection in peripheral blood between BLT mice, humans, and rhesus macaques (infected with R5-tropic SHIV), including plasma viremia and CD4^+^ T cell depletion from peripheral blood [32–34]. Perhaps more important, these data demonstrate that pre-exposure prophylaxis affords complete protection to humanized BLT mice from vaginal HIV-1 transmission.

**Figure 3:**
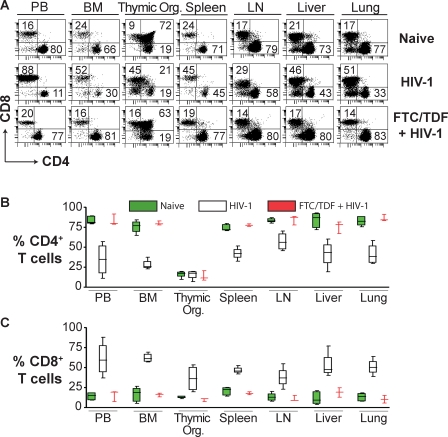
Systemic CD4^+^ T Cell Loss Resulting from Intravaginal HIV-1 Infection in Humanized BLT Mice (A) Comparison of the levels of CD4^+^ or CD8^+^ human T cells in the indicated tissues in representative BLT mice that were either naive, HIV-1 infected, or that received FTC/TDF for pre-exposure prophylaxis prior to exposure to HIV-1. Note the HIV-1 induced reduction in the double-positive CD4^+^CD8^+^ thymocytes. (B and C) Box plots depicting the levels of CD4^+^ (B) or CD8^+^ (C) T cells in the indicated tissues for naive (green), HIV-1 infected (white), and FTC/TDF-treated plus HIV-1–exposed (red) BLT mice. In these plots, the boxes extend from the first to the third quartiles, enclosing the middle 50% of the data. The middle line within each box indicates the median of the data, whereas the vertical line extends from lowest to the highest values. Data from naive, HIV-1-, or FTC/TDF-treated plus HIV-1–exposed mice were not collected on the same day. Naive (*n* = 5), HIV-1 infected (*n* = 4), and FTC/TDF + HIV-1 (*n* = 3). Flow cytometry gating for this figure was performed as described for Figure 2. BM, bone marrow; LN, lymph node; PB, peripheral blood; Thymic Org., implanted thymic organoid.

The systemic effects of HIV-1 infection in humans are inherently difficult to study. Therefore, we took advantage of the systemic repopulation of BLT mice with human lymphocytes to evaluate the effects of HIV-1 infection in relevant internal organs. Since CD4^+^ T cell depletion is a hallmark of HIV-1 infection, we compared the levels of these cells throughout the body of naive versus HIV-1–infected versus pre-exposure FTC/TDF-treated BLT mice. No statistical difference was observed when CD4^+^ T cell levels of all tissues combined in naive and pre-exposure FTC/TDF-treated BLT mice were compared (% *Mean_1_* − % *Mean_2_* [*M_1_* − *M_2_*] = 1.2 ± 8.8, *t* = 0.13, *df* = 65, *p* = 0.90). However, when comparing HIV-1–infected versus FTC/TDF-treated and HIV-1–exposed mice, statistically significant reductions in CD4^+^ T cells were noted in peripheral blood (*M_1_* − *M_2_* = −49 ± 13, *t* = 3.8, *df* = 5, *p* = 0.012), bone marrow (*M_1_* − *M_2_* = −52 ± 4.1, *t* = 13, *df* = 5, *p* < 0.001), spleen (*M_1_* − *M_2_* = −36 ± 4.7, *t* = 7.5, *df* = 5, *p* < 0.001), lymph nodes (*M_1_* − *M_2_* = −28 ± 7.4, *t* = 3.7, *df* = 5, *p* = 0.013), liver (*M_1_* − *M_2_* = −34 ± 11, *t* = 3.2, *df* = 5, *p* = 0.024), and lung (*M_1_* − *M_2_* = −45 ± 8.1, *t* = 5.6, *df* = 5, *p* = 0.003) in HIV-1–infected mice; no significant difference was noted in the thymic organoid (*M_1_* − *M_2_* = 1.8 ± 4.5, *t* = 0.40, *df* = 5, *p* = 0.70) (Figure 3A and 3B). Together with the reduction in the levels of CD4^+^ human T cells, there was a concomitant statistically significant increase in the levels of CD8^+^ human T cells comparing HIV-1–infected versus FTC/TDF-treated and HIV-1–exposed mice in all tissues tested, including peripheral blood (*M_1_* − *M_2_* = 45 ± 13, *t* = 3.4, *df* = 5, *p* = 0.019), bone marrow (*M_1_* − *M_2_* = 46 ± 3.5, *t* = 13, *df* = 5, *p* < 0.001), thymic organoid (*M_1_* − *M_2_* = 26 ± 9.9, *t* = 2.7, *df* = 5, *p* = 0.045), spleen (*M_1_* − *M_2_* = 29 ± 2.8, *t* = 10, *df* = 5, *p* < 0.001), lymph nodes (*M_1_* − *M_2_* = 27 ± 7.8, *t* = 3.4, *df* = 5, *p* = 0.019), liver (*M_1_* − *M_2_* = 34 ± 10, *t* = 3.4, *df* = 5, *p* = 0.019), and lung (*M_1_* − *M_2_* = 40 ± 6.5, *t* = 6.2, *df* = 5, *p* = 0.002) (Figure 3A and 3C).

**Figure 4:**
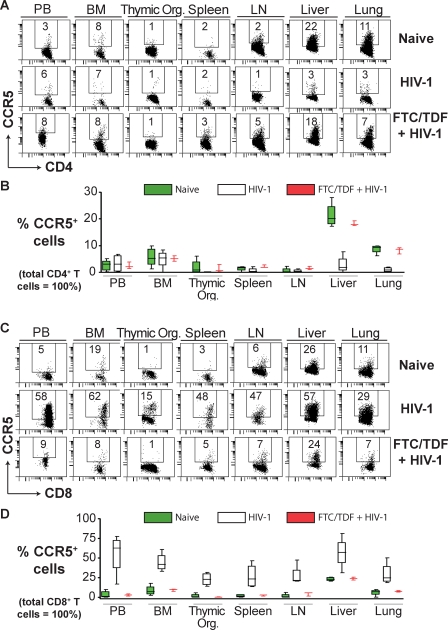
Changes in CD4^+^CCR5^+^ and CD8^+^CCR5^+^ Human T Cell Levels Resulting from HIV-1 Infection (A) Comparison of the levels of human CD4^+^CCR5^+^ T cells in the indicated tissues in a representative naive BLT mouse, an HIV-1–infected, and an HIV-1–exposed BLT mouse that received FTC/TDF for pre-exposure prophylaxis. Liver and lung were the examined tissues with the greatest constitutive CCR5 expression, and they both showed significant loss of CD4^+^CCR5^+^ T cells due to HIV-1 infection. (B) Box plot depicting the levels of CD8^+^CCR5^+^ T cells in the indicated tissues for naive (green), HIV-1 infected (white), and FTC/TDF-treated plus HIV-1–exposed (red) BLT mice. (C) Comparison of the levels of human CD8^+^CCR5^+^ T cells in the indicated tissues in representative naive, HIV-1 infected and FTC/TDF treated BLT mice. All tissues examined showed increases in CD8^+^CCR5^+^ T cells resulting from HIV-1 infection of BLT mice. (D) Box plot depicting the levels of CD8^+^CCR5^+^ T cells in the indicated tissues for naive (green), HIV-1–infected (white), and FTC/TDF-treated plus HIV-1–exposed (red) BLT mice. In the box plots, the boxes extend from the first to the third quartiles, enclosing the middle 50% of the data. The middle line within each box indicates the median of the data, whereas the vertical line extends from lowest to the highest values. Naive (*n* = 5), HIV-1 infected (*n* = 4), and FTC/TDF + HIV-1 (*n* = 3). Gating strategy for flow cytometric analysis: live cells → human CD45 → human CD3 → human CD4 or CD8 → CCR5. BM, bone marrow; LN, lymph node; PB, peripheral blood; Thymic Org., implanted thymic organoid.

CCR5 coreceptor expression levels on human lymphocytes vary by tissue, with lower levels on peripheral blood, bone marrow, thymus, spleen, and lymph node lymphocytes and higher levels in liver, lung, and GALT [16,35–37]. Comparison of HIV-1–infected versus FTC/TDF-treated and HIV-1–exposed mice demonstrated a significant reduction of CD4^+^CCR5^+^ T cells in BLT liver (*M_1_* − *M_2_* = −15 ± 2.1, *t* = 7.5, *df* = 5, *p* < 0.001) and lungs (*M_1_* − *M_2_* = −7.3 ± 0.77, *t* = 9.4, *df* = 5, *p* < 0.001); no significant difference was noted in the peripheral blood (*M_1_* − *M_2_* = 0.83 ± 2.1, *t* = 0.40, *df* = 5, *p* = 0.71), bone marrow (*M_1_* − *M_2_* = −0.33 ± 2.1, *t* = 0.16, *df* = 5, *p* = 0.88), thymic organoid (*M_1_* − *M_2_* = −1.1 ± 0.73, *t* = 1.5, *df* = 5, *p* = 0.19), spleen (*M_1_* − *M_2_* = −1.4 ± 0.59, *t* = 2.4, *df* = 5, *p* = 0.060), or lymph nodes (*M_1_* − *M_2_* = −1.1 ± 0.47, *t* = 2.3, *df* = 5, *p* = 0.069) (Figure 4A and 4B). We also observed a dramatic increase, indicative of a heightened state of immune activation, in the levels of human CD8^+^CCR5^+^ T cells in all tissues in response to HIV-1 infection between HIV-1–infected versus FTC/TDF-treated and HIV-1–exposed mice in peripheral blood (*M_1_* − *M_2_* = 52 ± 16, *t* = 3.3, *df* = 5, *p* = 0.022), bone marrow (*M_1_* − *M_2_* = 35 ± 7.2, *t* = 4.9, *df* = 5, *p* = 0.005), thymic organoid (*M_1_* − *M_2_* = 23 ± 5.0, *t* = 4.5, *df* = 5, *p* = 0.006), spleen (*M_1_* − *M_2_* = 24 ± 9.3, *t* = 2.6, *df* = 5, *p* = 0.048), lymph nodes (*M_1_* − *M_2_* = 23 ± 7.8, *t* = 3.0, *df* = 5, *p* = 0.031), liver (*M_1_* − *M_2_* = 33 ± 12, *t* = 2.7, *df* = 5, *p* = 0.043), and lung (*M_1_* − *M_2_* = 22 ± 8.5, *t* = 2.6, *df* = 5, *p* = 0.050) (Figure 4C and 4D). Thus, HIV-1 infection altered the proportions of CCR5^+^ T lymphocytes throughout BLT mice. Together, these results demonstrate that intravaginal HIV-1 transmission in humanized BLT mice leads to systemic effects by the virus that closely mimics what is observed in infected humans.

**Figure 5:**
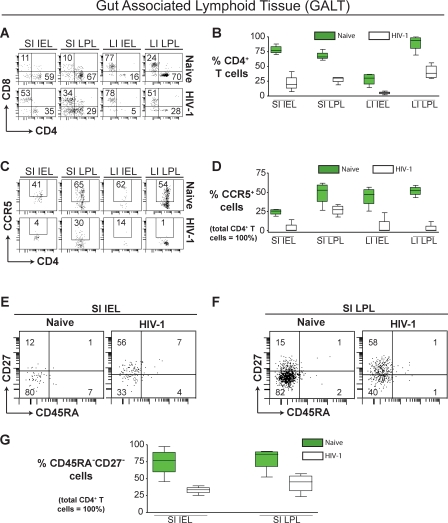
Loss of CCR5^+^ and Effector Memory CD4^+^ T Cells from GALT during HIV-1 Infection (A) Comparison of the levels of CD4^+^ or CD8^+^ human T cells in the GALT of representative naive and HIV-1–infected mice. (B) Box plot depicting the levels of GALT CD4^+^ T cells for naive (green) and HIV-1–infected (white) BLT mice. (C) Comparison of the levels of human CD4^+^CCR5^+^ T cells in the GALT of naive and HIV-1–infected BLT mice. Significantly fewer GALT CD4^+^ T cells had detectable CCR5 expression levels following HIV-1 infection. (D) Box plot depicting the levels of CD4^+^CCR5^+^ T cells in the GALT for naive (green) and HIV-1–infected (white) BLT mice. (E and F) Comparison of the levels of human CD4^+^ effector memory T cells in the small intestine intra-epithelial (E) and lamina propria (F) lymphocyte compartments of representative naive and HIV-1–infected BLT mice. HIV-1–infected BLT mice have statistically fewer effector memory CD4^+^ T cells present in their small intestines. (G) Box plot depicting the levels of CD45RA^neg^CD27^neg^ effector memory T cells in the small intestine of naive (green) and HIV-1–infected (white) BLT mice. In the box plots, the boxes extend from the first to the third quartiles, enclosing the middle 50% of the data. The middle line within each box indicates the median of the data, whereas the vertical line extends from lowest to the highest values. Naive (*n* = 4), HIV-1 infected (*n* = 4). Gating strategy for flow cytometric analysis: live cells → human CD45 → human CD3 → human CD4 → CCR5, CD27, or CD45RA. IEL, intraepithelial lymphocytes; LI large intestine; LPL, lamina propria lymphocytes; SI, small intestine.

The GALT is a major site of HIV-1 replication and CD4^+^ T cell depletion during HIV disease in humans [38]. Therefore, we isolated intraepithelial (IEL) and lamina propria (LPL) lymphocytes from both small and large intestine (SI and LI, respectively) of BLT mice for analysis. Consistent with what has been observed during the course of human HIV-1 infection, we also observed a dramatic reduction in CD4^+^ T cells in the SI IEL (*M_1_* − *M_2_* = 56 ± 8.0, *t* = 7.0, *df* = 6, *p* < 0.001), SI LPL (*M_1_* − *M_2_* = 40 ± 4.9, *t* = 8.3, *df* = 6, *p* < 0.001), LI IEL (*M_1_* − *M_2_* = 23 ± 5.3, *t* = 4.3, *df* = 6, *p* = 0.005), and LI LPL (*M_1_* − *M_2_* = 49 ± 9.3, *t* = 5.2, *df* = 6, *p* = 0.002) (Figure 5A and 5B). As described above for liver and lung, HIV-1 infection resulted in a significant reduction of CD4^+^CCR5^+^ T cells in BLT mouse SI IEL (*M_1_* − *M_2_* = 21 ± 4.1, *t* = 5.1, *df* = 6, *p* = 0.002), SI LPL (*M_1_* − *M_2_* = 22 ± 8.5, *t* = 2.6, *df* = 6, *p* = 0.042), LI IEL (*M_1_* − *M_2_* = 38 ± 8.9, *t* = 4.3, *df* = 6, *p* = 0.005), and LI LPL (*M_1_* − *M_2_* = 48 ± 4.4, *t* = 11, *df* = 6, *p* < 0.001) (Figure 5C and 5D). We also observed a statistically significant reduction in the levels of CD4^+^ effector memory T cells (CD45RA^neg^CD27^neg^) from the SI IEL (*M_1_* − *M_2_* = 41 ± 11, *t* = 3.7, *df* = 6, *p* = 0.010) and SI LPL (*M_1_* − *M_2_* = 36 ± 12, *t* = 3.1, *df* = 6, *p* = 0.021) of infected BLT mice (Figure 5E–5G). These findings are in agreement with studies in humans and macaques regarding memory T cell loss in GALT by HIV-1 and SIV/SHIV [24,38–41], and highlight the usefulness of the BLT model for studying HIV-1 pathogenesis, particularly in GALT.

**Figure 6:**
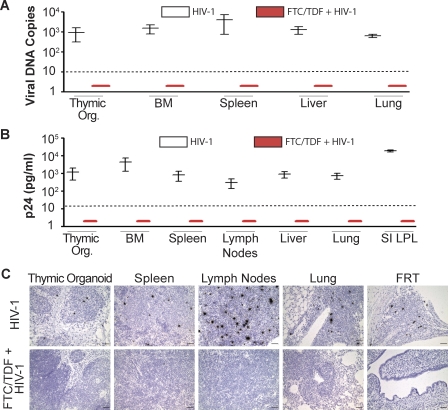
Pre-exposure FTC/TDF Treatment Resulted in Complete Protection of Humanized BLT Mice from Intravaginal HIV-1 Transmission (A) Box plot depicting real-time PCR levels of HIV-1 viral DNA in the indicated tissues for HIV-1–infected (white) and FTC/TDF-treated plus HIV-1–exposed (red) BLT mice. (Viral DNA copies per million CD4^+^ T cells shown.) HIV-1 infected (*n* = 2) and FTC/TDF + HIV-1 (*n* = 4). (B) Box plot depicting virus rescue results from HIV-1–infected (white) and FTC/TDF-treated plus HIV-1–exposed (red) BLT mice. Virus rescue data expressed as pg/ml of p24 per 1 × 10^5^ CD4^+^ T cells cocultured with PHA/IL2-activated peripheral blood lymphocyte from a seronegative donor. HIV-1 infected (*n* = 2) and FTC/TDF + HIV-1 (*n* = 4). In the box plots, the middle line indicates the median of the data, whereas the vertical line extends from lowest to the highest values. Dashed lines indicate the limit of detection for each assay. (C) In situ hybridization analysis to determine the presence of productively HIV-1–infected cells in the indicated tissues from HIV-1–infected or FTC/TDF-treated BLT mice (bars indicate 50 μm). Note the lack of HIV-1 in the BLT mice that received pre-exposure prophylaxis with FTC/TDF. HIV-1 infected (*n* = 4) and FTC/TDF + HIV-1 (*n* = 3). BM, bone marrow; LPL, lamina propria lymphocytes; Thymic Org., implanted thymic organoid; SI, small intestine.

Finally, to confirm the lack of infection in FTC/TDF-treated animals shown in Figures 2, 3, and 4, three additional approaches were utilized. DNA isolated from cells obtained from different organs of either HIV-1–infected or FTC/TDF-treated BLT mice was analyzed by quantitative real-time PCR for HIV-1 viral DNA. Whereas the tissues from HIV-1–infected mice were clearly positive for viral DNA, samples from pre-exposure FTC/TDF-treated mice were consistently negative (Figure 6A). Cells isolated from multiple organs of HIV-1–infected or FTC/TDF-treated BLT mice were cocultured with PHA/IL2-activated peripheral blood lymphocytes from a seronegative donor. Virus was readily rescued from cells isolated from tissues obtained from the HIV-1–infected mice (Figure 6B). In contrast, no virus was rescued from any of the tissues obtained from the BLT mice treated with FTC/TDF. Last, we used in situ hybridization to determine the presence of productively infected cells in HIV-1–infected or FTC/TDF-treated BLT mice. Productively HIV-1–infected cells were readily observed in tissues from the HIV-1–infected BLT mice (Figure 6C). In contrast, no productively infected cells were found in any of the tissues from the FTC/TDF-treated mice. These results verify the protection afforded by this pre-exposure prophylaxis approach to the prevention of intravaginal transmission of HIV-1 in BLT mice.

## Discussion

The present study demonstrates efficient intravaginal HIV-1 transmission in humanized BLT mice that results in a systemic reduction of engrafted human CD4^+^ T cells and a loss of GALT effector memory human CD4^+^ T cells, as has been observed in humans [24,38–41]. In addition, we provide evidence of the effectiveness of antiretrovirals for pre-exposure prophylaxis to prevent intravaginal HIV-1 transmission.

In the absence of an effective vaccine or topical microbicide, alternative preventative measures are desperately needed to help block the spread of AIDS. Antiretroviral drugs have considerable potential for preventing HIV-1 transmission [42]. The expectation for pre-exposure prophylaxis is that antiretroviral drugs taken appropriately can prevent HIV infection [4]. There is as yet no clinical evidence for the effectiveness of this approach [43–46]. However, precedent for the administration of antiretrovirals to large populations of individuals at high risk for infection is exemplified by the widespread use of nevirapine for the prevention of mother-to-child transmission of HIV [47,48]. Similarly, if proven safe and effective, pre-exposure prophylaxis together with other behavioral interventions could provide protection to men and women at risk of HIV infection by preventing sexual transmission. Therefore, it is critical to evaluate new prevention methods aimed at the populations at highest risk. Despite the urgency to develop and implement novel approaches capable of preventing HIV transmission, this process has been hindered by the lack of adequate animal models readily available for pre-clinical efficacy and safety testing [49].

We investigated the possibility that BLT mice might serve as an efficient, relatively fast, and cost-effective small animal model of intravaginal HIV-1 infection. We demonstrate that the female reproductive tract of BLT mice is populated with in situ–generated human cells critical for the transmission and dissemination of HIV-1. We observed that a single intravaginal exposure to HIV-1 results in infection in 88% of the exposed humanized BLT mice, demonstrating their susceptibility to vaginal transmission. These observations distinguish the BLT system (with its self-renewing, hematopoietic stem cell–based systemic human reconstitution, including throughout the female reproductive tract) from SCID mice injected with human peripheral blood lymphocyte (SCID-hu PBL) with respect to vaginal HIV-1 transmission [50,51]. The systemic nature of BLT human reconstitution facilitated examination of the pathogenic effects caused by infection in BLT mice. Our analyses revealed that HIV-1 disseminates from the vaginal mucosa to cause systemic CD4^+^ T cell loss, including GALT CD4^+^ effector memory T cell loss, as in humans [38]. Thus, humanized BLT mice represent a useful model for HIV-1 intravaginal transmission, systemic spread, and pathogenesis.

We utilized the fact that BLT mice are susceptible to intravaginal HIV-1 infection to demonstrate that this system is well suited for the preclinical evaluation of pre-exposure prophylactic regimens to prevent intravaginal HIV-1 transmission. Our results show that the BLT model can serve as a relatively fast and simple system to test whether pre-exposure prophylaxis can prevent vaginal HIV-1 transmission. Using this system, we found that FTC/TDF can afford complete protection from vaginal HIV-1 transmission. These results suggest that the BLT model could also be suitable for testing topical microbicides. Our results serve as preclinical evidence for the potential success of this approach aimed at preventing the further spread of AIDS.

As with all animal models of human disease, there are limitations to this study. Although our findings are consistent with findings from non-human primate research regarding the potential of pre-exposure prophylaxis to prevent HIV-1 transmission [2,42], neither model has been shown to predict efficacy or safety in humans. This is due to the lack of any kind of data from similar pre-exposure prophylaxis in humans. Therefore, an important limitation is that the BLT model currently has no known predictive value for clinical medicine. It is essential that this and future BLT studies be validated against data from human clinical trials, some of which are ongoing. Variables between humanized BLT mice and humans include possible differences in drug concentrations, in adherence, in renal and liver biology, virus dosage, and coinfections with viruses such as hepatitis B virus. Although many aspects of HIV-1 GALT pathogenesis are recapitulated in BLT mice, we have not determined whether there is a direct and/or indirect pathologic effect of HIV-1 on enterocytes, as seen in humans. Many of these limitations can be addressed in future studies. In the interim, our data support the potential for antiretrovirals in general and FTC/TDF in particular to function as a pre-exposure prophylaxis measure against the spread of HIV/AIDS in humans.

More women are being infected by HIV-1 now than at any other time during the AIDS epidemic. The number of infected women worldwide has increased to almost 15.4 [1]. As a female-controlled prevention measure, antiretroviral pre-exposure prophylaxis and/or topical microbicides could provide women with a powerful tool to protect themselves from infection. However, any candidate drug(s) must be safe, especially in individuals without disease, and efficacious and, in order to be successful, must be easy to use [4]. The combination of FTC/TDF appears to meet the criteria for drugs to be used for pre-exposure prophylaxis [31]. In addition, it is one of the few drug combinations that can be administered once daily without food restrictions. In this report, we provide preclinical evidence regarding the potential efficacy of antiretroviral pre-exposure prophylaxis in humans. Our results should provide further impetus for the continued implementation of clinical trials using oral antiretroviral pre-exposure prophylaxis, particularly in parts of the world with highest HIV prevalence, where pre-exposure prophylaxis would be most beneficial and cost effective.

## Abbreviations

BLT: bone marrow–liver–thymus
FRT: female reproductive tract
FTC: emtricitabine
GALT: gut-associated lymphoid tissue
HSC: hematopoietic stem cell
IEL: intraepithelial lymphocytes
LI: large intestine
LPL: lamina propria lymphocytes
SHIV: chimeric simian–human immunodeficiency virus
SI: small intestine
TDF: tenofovir disoproxil fumarate
